# Unraveling the mechanisms of NK cell dysfunction in aging and Alzheimer’s disease: insights from GWAS and single-cell transcriptomics

**DOI:** 10.3389/fimmu.2024.1360687

**Published:** 2024-02-23

**Authors:** Jinwei Li, Yang Zhang, Yanwei You, Zhiwei Huang, Liya Wu, Cong Liang, Baohui Weng, Liya Pan, Yan Huang, Yushen Huang, Mengqi Yang, Mengting Lu, Rui Li, Xianlei Yan, Quan Liu, Shan Deng

**Affiliations:** ^1^ Department of Neurosurgery, Liuzhou Workers Hospital, Liuzhou, China; ^2^ Department of Neurosurgery, West China Hospital, Sichuan University, Chengdu, Sichuan, China; ^3^ Department of Vascular Surgery, Fuwai Yunnan Cardiovascular Hospital, Affiliated Cardiovascular Hospital of Kunming Medical University, Kunming, Yunnan, China; ^4^ Division of Sports Science and Physical Education, Tsinghua University, Beijing, China; ^5^ Department of Neurology, Liuzhou Workers Hospital, Liuzhou, China; ^6^ Department of Pharmacy, Liuzhou Workers Hospital, Liuzhou, China; ^7^ Department of Dermatology, Liuzhou Workers Hospital, Liuzhou, China; ^8^ Department of Medical Imaging, Liuzhou Workers Hospital, Liuzhou, China

**Keywords:** AD, aging, ScRNA-seq, Mendelian randomization, NHANES

## Abstract

**Background:**

Aging is an important factor in the development of Alzheimer’s disease (AD). The senescent cells can be recognized and removed by NK cells. However, NK cell function is gradually inactivated with age. Therefore, this study used senescence as an entry point to investigate how NK cells affect AD.

**Methods:**

The study validated the correlation between cognition and aging through a prospective cohort of the National Health and Nutrition Examination Survey database. A cellular trajectory analysis of the aging population was performed using single-cell nuclear transcriptome sequencing data from patients with AD and different ages. The genome-wide association study (GWAS) cohort of AD patients was used as the outcome event, and the expression quantitative trait locus was used as an instrumental variable. Causal associations between genes and AD were analyzed by bidirectional Mendelian randomization (MR) and co-localization. Finally, clinical cohorts were constructed to validate the expression of key genes.

**Results:**

A correlation between cognition and aging was demonstrated using 2,171 older adults over 60 years of age. Gene regulation analysis revealed that most of the highly active transcription factors were concentrated in the NK cell subpopulation of AD. NK cell trajectories were constructed for different age populations. MR and co-localization analyses revealed that *CHD6* may be one of the factors influencing AD.

**Conclusion:**

We explored different levels of AD and aging from population cohorts, single-cell data, and GWAS cohorts and found that there may be some correlations of NK cells between aging and AD. It also provides some basis for potential causation.

## 1 Introduction

With the aging of the population, dementia has become a common disease among the elderly. Alzheimer’s disease (AD) dementia accounts for 60% to 80%, and AD patients will reach 152 million by 2050 (1). In addition, people with Alzheimer’s disease experience a gradual decline in cognitive abilities, leading to loss of language skills, learning difficulties, memory loss, and personality and mood changes (2). However, AD prevention and treatment are a worldwide challenge. There is an urgent need to find biological markers for early diagnosis, differential diagnosis, and early outcome prediction.

Aging is the biggest risk factor for almost all major chronic diseases. Moreover, aging is characterized by the gradual loss of physiological integrity, resulting in impaired function and increased susceptibility to death. This deterioration is a major risk factor for human pathology, including cancer, diabetes, cardiovascular disease, and neurodegenerative diseases (3). In addition, studies have shown that aging is a key risk factor for AD. Some studies have shown that senescent cells accumulate in tissues and lead to age-related pathological changes by releasing inflammatory factors (4). Therefore, it is very important to understand the relationship between aging and AD and the related mechanisms.

When it comes to AD, the immune system plays a crucial role in the development and progression of the disease (5, 6). Tau protein serves as an important AD-specific biomarker. The number of T cells, particularly cytotoxic T cells, significantly increases in the tau pathology regions of tau transgenic mice and AD brains (7). By integrating single-cell RNA sequencing (scRNA-seq) and single-cell T cell receptor (TCR) sequencing (scTCR-seq) analysis, this study demonstrated the association of CD8 T cells with age-dependent accumulation of disease in the brain parenchyma (8). The application of multi-omics studies in AD patients can provide comprehensive molecular-level information, assisting researchers in gaining a deeper understanding of the pathogenesis of AD and identifying potential biomarkers (9–12). AD patients exhibit multiple cellular subpopulations in their blood, which may play different roles in the development and progression of the disease (13). Bioinformatics-based scRNA-seq can aid researchers in analyzing and comparing the cellular subpopulations in the blood of AD patients (14). The gene regulatory network (GRN) determines and maintains the cell-type-specific transcriptional states, which, in turn, form the basis of cellular morphology and function (15). Therefore, it is crucial to identify the GRN of the cell subpopulations associated with the pathogenesis of AD and further investigate their functions and interactions.

Recent studies have shown that NK cells are the core participants in the monitoring of aging cellular immunity. With age, NK cell dysfunction is associated with an increased burden of infection, malignant tumors, inflammatory diseases, and senescent cells (16). NK cells can help clear α-synuclein, reduce inflammation produced by autologous active T cells, and clear damaged neurons. NK cells are essential for regulating and inhibiting inflammation and abnormal protein accumulation in brain tissue (17). The expanded and cultured autologous NK cells not only showed a strong ability to kill tumor cells and scavenge senescent cells but also reduced the senescence markers of peripheral blood mononuclear cells (PBMC) in blood after reinfusion (18). NKGen Biotech recently presented data from its phase 1 clinical trial of NK cell therapy SNK01 in patients with Alzheimer’s disease at the Alzheimer’s Association International Conference. The functional score of 70% of the patients remained stable or improved in the 11th week. As the key to non-invasive diagnosis, human blood reflects the physiological and pathological status of patients to a certain extent. Some studies have explored the key role of peripheral blood in the diagnosis of AD (13, 19, 20). Therefore, it is necessary to further explore the NK cells in aging and AD patients.

We further explore the biomarkers of early diagnosis and early prediction of curative effect and provide a theoretical basis for the study of AD in elderly patients. A large sample of the National Health and Nutrition Examination Survey (NHANES) database was used to prospectively evaluate the correlation between cognitive impairment and aging. Moreover, scRNA-seq from normal patients, AD, umbilical cord blood, and young and aging patients was used to explore the developmental trajectory of NK cells in different age groups, combined with high transcriptional activity genes in transcriptional gene regulation in AD patients to construct aging cytokinetic clusters. In addition, combined with expression quantitative trait locus (eQTLs) data, two-way Mendelian randomization (MR) and Bayesian co-location analysis were used to explore the causal relationship between senescence genes and AD in NK cells.

## 2 Method

### 2.1 Data source and processing

The study group in this study met the clinical diagnostic criteria established by the National Institute on Aging and the Alzheimer’s Disease Association in 2011, which consisted of (1) meeting the diagnostic criteria for dementia, (2) having an insidious onset, with symptoms appearing progressively over months to years, (3) having a clear history of cognitive impairment, and (4) presenting with amnestic syndrome (decline in learning and near-memory with impairment in one or more other cognitive domains) or non-amnesic syndrome (impairment of one of the three domains of language, visuospatial, or executive functioning, accompanied by impairment in one or more other cognitive domains). The control group was selected from healthy adults who underwent physical examination at the Liuzhou Workers’ Hospital Physical Examination Center (2). A total of four AD patients and four normal patients were selected. The study was approved by the Institutional Ethics Committee and Institutional Review Board of Liuzhou Workers’ Hospital (Ethics Code: KY2023140), and all participants signed an informed consent form.

Single-cell datasets were collected from the Gene Expression Omnibus (GEO) (https://www.ncbi.nlm.nih.gov/geo/) database, including four patients with AD (two early and two late) and two normal controls (GSE168522) as well as three cord blood samples, three healthy young people, and six healthy aging participants (GSE157007). The aging participants were defined as 60 years or older. The AD patients fulfilled the following inclusion criteria: a clinical diagnosis of mild or severe AD, a Mini-Mental State Examination (MMSE) score >19, an age range of 60–90 years, and stable administration of anti-dementia or mood-stabilizing medication (21). All of these patients had a neurologist’s diagnosis. The quality control process was as follows: set the number of genes and ribosomal ratio minGene = 200, maxGene = 3,000, and pctMT = 5. A total of 2,000 high variants are selected for the following analysis, and each sample is carried out in batches using the R package “harmony”. After clustering, the cell clusters were annotated by manual annotation as follows: CD4 (“CD3E”, “IL7R”, and “CD4”), NK (“NKG7”, “KLRB1”, and “CCL5”), Mac (“CD68” and “MARCO”), CD8 (“CD8A”), B (“CD79A”, “CD19”, and “MS4A1”), Mono (“CD14”, “S100A9”, and “S100A12”), mDC (“CD1C”, “FCER1A”, and “CLEC9A”), pDC (“JCHAIN”, “CLEC4C”, and “LILRA4”), and plasma (“IGHG1” and “IGKC”). The maker of cell annotation comes from CellMarker and related literature (13, 22, 23).

Transcriptome data were obtained from the GEO dataset (GSE63060 and GSE63061) with 139 AD patients and 238 normal patients. Dataset GSE63060 and dataset GSE63060 were merged for analysis (24). The Combat method in the “sva” package performs batch corrections on the merged data.

GWAS data comes from public datasets called “ieu-b-5067” and “ebi-a-GCST90027158”. In the ieu-b-5067 dataset, there were 954 AD cases and 487,331 healthy people. The number of AD cases in the verified dataset ebi-a-GCST90027158 was 39,106 and that of healthy persons was 46,828. The eQTLs are a kind of genetic loci that can affect gene expression; most of them are single-nucleotide polymorphisms (SNP), which have a certain biological significance. The eQTLs data of genes were obtained from the IEU database (https://gwas.mrcieu.ac.uk/).

The present prospective cohort investigation was predicated on an analysis of openly accessible and identified data sourced from the NHANES. Consequently, no supplementary institutional review board authorization or explicit informed consent was required. The NHANES constituted a comprehensive nationwide survey encompassing civilian, non-institutionalized respondents within the United States. This annual survey was administered by the National Center for Health Statistics, an entity under the aegis of the Centers for Disease Control and Prevention. Detailed elucidation regarding ethical clearances and procedures for informed consent can be obtained from the NHANES website (https://wwwn.cdc.gov/Nchs/Nhanes/). Comprehensive insights into the NHANES framework, methodology, and weighting schema have been expounded elsewhere. In succinct terms, the NHANES adopts a multifaceted stratified sampling methodology, characterized by a complex multistage design, for household selection from randomly allocated clusters.

In the initial cohort of NHANES 1999–2002, 3,234 individuals aged 60 and above were enrolled in the study. Subsequently, those lacking cognitive assessment data (*n* = 298) were omitted, resulting in a subsample of 2,936. Further refinement involved the exclusion of participants without requisite information for phenotypic age (PhenoAge) calculation (*n* = 212), yielding a subsample of 2,724. Lastly, 553 individuals without complete covariate data were excluded from the analytical cohort, culminating in a final study population of 2,171 individuals.

### 2.2 Measurement of PhenoAge and cognitive test

The emergence of a novel phenotypic age metric, as opposed to reliance on chronological age in isolation, emerges as a pivotal advancement in forecasting aging-related prognoses. Drawing from established literature (25–27), an array of 10 variables, encompassing metrics such as chronological age and albumin level, were employed in the computation of PhenoAge. This metric is precisely defined by the following formula:


$$\text{Phenotypic age}=141.50\;+\;\frac{\text{Ln}\left[-0.00553\times \text{Ln}\left(\exp\left(\;\frac{-1.51714\times \exp\left(xb\right)}{0.0076927}\right)\right)\right]}{0.09165}$$

$$\begin{array}{l} xb=-19.907-0.0336\times albumin\left(g/L\right)+0.0095\times creatinine\left(umol/L\right)\\ +0.1953\times glucose\left(mmol/L\right)+0.0954\times ln\left(CRP\right)\left(mg/dl\right)\\ -\;0.0120\times lymphocyte\;percent\left(\%\right)+0.0268\\ \times \;mean\;cell\;volume\left(fL\right)+0.3306\\ \times \;red\;cell\;distribution\;width\left(\%\right)+0.00188\\ \times \;alkaline\;phosphatase\left(U/L\right)+0.0554\\ \times \;white\;blood\;cell\;count\left(1000cell/uL\right)+0.0804\\ \times \;chronological\;age\left(years\right) \end{array}$$

In consonance with precedent investigations utilizing the NHANES datasets, cognitive function was assessed by employing the Digit Symbol Substitution test (DSST) (28, 29). The DSST, an integral module of the Wechsler Adult Intelligence Scale (WAIS-III), gauges processing speed, sustained attention, and working memory capacities. This assessment tool has found wide-ranging applications in diverse contexts, spanning extensive screenings, epidemiological surveys, and clinical appraisals. Administered during the household interviews in NHANES 1999–2002, the exercise entails the use of a paper form featuring a key section at the apex, enumerating nine numbers each concomitantly paired with symbols. The participants are allocated 2 min to replicate the corresponding symbols in the 133 adjoining boxes alongside the numbers. The score derives from the aggregate of correct matches accomplished.

### 2.3 Covariate assessment statistical analysis

In the present study, covariates encompassed sex, race, body mass index, education, marital status, poverty status, and prevalent chronic disease conditions, including cardiovascular diseases, hypertension, and diabetes mellitus. The analytical procedures appropriately integrated NHANES sampling weights, duly accounting for the intricate multistage cluster survey design. Weighted linear regression models were employed to discern the relationship between PhenoAge and cognitive function test outcomes. Consistent with established research paradigms (30–32), both multivariate adjusted and unadjusted models were applied: The crude model entailed no covariate adjustments. Model 1 was calibrated for sex and race, while model 2 accounted for sex, race, body mass index, education, marital status, poverty status, and chronic disease conditions. All statistical analyses adhered to the methodological guidelines outlined by the Centers for Disease Control and Prevention (33). Significance was denoted by *P*< 0.05. The entire analytical framework was executed using R version 4.2.0 (http://www.R-project.org, The R Foundation).

### 2.4 Identification of transcription factor gene regulatory network in AD

Firstly, AD and normal meta-cell matrices were constructed to make it clear that transcription factors drive transcriptome changes in patients with AD (34). The transcription factor gene set from the reference genomic hg38 of cistarget was defined (https://resources.aertslab.org/cistarget/tf_lists/allTFs_hg38.txt). To filter the low-expression genes, the standard is mc. mat > 0pr. In. Cells > = 5. Genes that are not in cisTargetDB (35) were filtered. To construct the gene regulatory network of AD and normal people, filter the regulon and set the minimum number of regulon genes. To identify the PBMC cell type with the greatest transcriptional activity in AD, the regulon activity score (RAS) matrix was calculated with AUCell (36). Uniform Manifold Approximation and Projection (UMAP) showed the results of “harmony”, “PCA”, and RAS. The comparison showed the activity of regulatory transcription factors related to cell type and AD. UMAP was constructed to classify cells by transcriptional activity and gene expression. The algorithm random forest inferred the co-expression module between transcription factors and candidate target genes (37–39). According to the clustering results, 9 was selected as the appropriate cut tree. The R packets “ComplexHeatmap” and “plot1cell” were used to show the clustering results. The dryness of AD cells was inferred by CytoTRACE.

### 2.5 Determine the timing trajectory of aging cells

To identify the developmental trajectory of NK cells in unicellular PBMC of umbilical cord blood, young, and aging, the force-directed graph (FDG) was compared with other scRNA-seq dimensionality reduction methods. Through the shortest path algorithm, the lineage of the young and the aging was constructed. The developmental branch plot was drawn, and the differential potential (DP) was calculated to visualize the location of the greatest change in DP and the maximum point of the gene in the elderly. Moreover, the metabolic time sequence of the red curve changed, and the blue curve represented the change in differentiation potential. To determine the relationship between different genes related to aging time series, we used k-nearest neighbor conditional density resampling mutual information estimation (KNN-DREMI). DREVIPlot was used for time series clustering, and specific methods to refer to Leizhang were used (40). The clustering cluster was set as 10. 0 through DREVI. Furthermore, cluster 3 was selected in the following Mendelian randomized analysis.

### 2.6 Cellular communication network in aging and AD

To further understand the network of NK cells and other cell subsets, we inferred the cellular communication network. We used a circle diagram to visualize the number of interactions or the total interaction intensity between cell subsets. The hierarchical structure map visualized the cell–cell communication regulated by a single ligand–receptor pair.

### 2.7 Mendelian randomization analysis

We aimed to evaluate the causal effect between the expression of NK aging-related genes and AD. In this study, we use “TwoSampleMR” (https://github.com/MRCIEU/TwoSampleMR) (v0.5.6) for MR analysis. To obtain independent SNPs, aggregation was performed to remove SNPs with linkage disequilibrium (LD) *R^2^* < 0.001. The traits were used at the genome-wide level (*p*< 5 × 10^-8^). The instrumental strength of each SNP was assessed using the F statistic = (β/SE). Mean F-statistics are reported for SNPs used as instruments, with F-statistics >10 indicating strong instruments (41). The main analysis is as follows: (1) SNPs as a tool variable (eQTLs), senescence track gene in NK cells as exposure, and AD as the outcome and (2) SNPs was a tool, AD was exposure, and senescence trajectory gene in NK cells is the outcome. Five MR methods (including MR Egger, weighted median, inverse variance weighted, simple model, and weighted model) are used for the robust analysis of causality. When there is only one tool variable, the Wald ratio is used to estimate the effect of exposure on the outcome. Finally, reverse causality is evaluated.

### 2.8 Bayesian co-localization analysis

In Bayesian co-mapping analysis, we used the “coloc” software package (https://github.com/chr1swallace/coloc) and default parameters to evaluate the probability of two traits sharing the same causal variant. Bayesian co-mapping provides *a posteriori* probability of five hypotheses about whether a single variant is shared between two traits (42). In this study, we tested the posterior probability of hypothesis 3 (PPH3) and hypothesis 4 (PPH4). In hypothesis 3, both pQTL and MS are associated with this region through different mutations; in hypothesis 4, both pQTL and MS are associated with this region through shared variation. We use coloc.abf and coloc.susie algorithms to define genes based on co-mapping evidence based on gene PPH4 > 50% determined by at least one algorithm (43).

### 2.9 Comparative transcriptomic and immune cell infiltration analysis of risk genes in AD

In AD transcriptional group sequencing, the “CIBERSORT” and “MCPcounter” R packets were used to analyze immune cell infiltration in AD and normal patients. At the same time, determining the difference in *CHD6* expression between AD and normal patients was carried out. The single-sample gene set enrichment analysis (ssGSEA) showed the correlation between *CHD6* and the signal pathway.

### 2.10 Peripheral blood mononuclear lymphocyte collection and pretreatment procedure

The anticoagulant tube blood sample was transferred to a 15-mL centrifuge tube, and PBS was added to 10 mL and mixed well. The mixing night was transferred to a 50-mL centrifuge tube containing 10 mL of lymphatic isolate, without mixing, so that the mixing night is in the upper layer of the lymphatic split, and it was centrifuged at 2,000 g/min for 20 min at 25°C. Taking the turbid intermediate layer into a 15-mL centrifuge tube, PBS was added to 10 mL and centrifuged again at 2,000 g/min for 10 min at 25°C. The supernatant was discarded, and desktop centrifugation was performed. Furthermore, 1 mL of erythrocyte lysate (Beijing Solepol)-purified PBMC added, blown to mix, and centrifuged at 1,500 g/min for 3 min at 25°C. The supernatant was discarded, and tabletop centrifugation proceeded. In addition, 1 mL trizol was added, blown to mix well, and transferred to 2-mL EP tubes. The whole process was carried out at room temperature.

### 2.11 RNA extraction and quantitative real-time polymerase reaction

Human peripheral blood mononuclear lymphocyte (PBMC) total RNA was extracted using TRIzol Reagent RNAiso plus Total RNA Extraction Reagent (Takara, Japan). The concentration and purity of the RNA samples were measured by using Nanodrop 2000 (Thermo Fisher Scientific, USA). Reverse transcription was performed using the PrimeScript TMRT Reagent Kit with gDNA Eraser with random primers and gDNA removal (Takara, Japan). Then, cDNA amplification was performed using TB Green Premix Ex Taq II (Takara, Japan) and Applied Biosystems 7500 Fast Real-time PCR System Sequence Detection System (Applied Biosystems, USA). GAPDH or β-actin was used as an internal reference, and each sample was repeated three times. The relative quantification of mRNA expression was compared with the internal reference and analyzed using the 2^-ΔΔCT^ method. The primer sequences are shown in **Supplementary Table S1**.

## 3 Results

### 3.1 Prospective cohort database to explore the correlation between cognitive performance and PhenoAge

The cohort comprised 2,171 individuals aged 60 years or older, reflecting a weighted population estimate of 31,235,578. The sociodemographic characteristics of the participants are delineated in **Table 1**. The mean PhenoAge, denominated in years, was 66.13 ± 0.34, with male patients constituting 45.12% of the sample. The Digit Symbol Substitution test, an indicator of processing speed, sustained attention, and working memory, yielded an average score of 47.19 ± 0.80. Utilizing a weighted linear regression model, the relationship between Digit Symbol Substitution test scores and PhenoAge was examined. When PhenoAge was treated as a continuous variable, the results presented in **Table 2** divulge a negative association with cognitive performance across all models [crude model, β (95% CI): -0.569 (-0.648, -0.490), *p*< 0.001; model 1, β (95% CI): -0.599 (-0.684, -0.513), *p*< 0.001; model 2, β (95% CI): -0.383 (-0.463, -0.303), *p*< 0.001]. Additionally, an association between PhenoAge, considered a categorical variable, and test scores was observed. In the fully adjusted model 2, with Q1 as the reference, Q2, Q3, and Q4 were all significantly correlated with test scores [Q2, β (95% CI): -4.053 (-5.942, -2.165), *p*< 0.001; Q3, β (95% CI): -8.304 (-10.544, -6.064), *p*< 0.001; Q4, β (95% CI): -12.937 (-15.135, -10.739), *p*< 0.001]. Furthermore, stratified analyses (as outlined in **Supplementary Table S2**) underscored the consistency of these associations across various subgroups.

**Table 1:** Demographic characteristics of study participants in National Health and Nutrition Examination Survey.

Variable	Value^a^
Age	
<65	26.73
(65–72)	34.67
≥72	38.60
Sex	
Male	45.12
Female	54.88
Race/ethnicity	
Non-Hispanic white	83.07
Non-Hispanic black	6.56
Mexican American	2.86
Other race/ethnicity	7.51
Marital status	
Never married	2.35
Married/living with partner	65.39
Widowed/divorced	32.26
Education	
Below high school	13.30
High school	46.13
College or above	40.57
Poverty income ratio	
<1	12.10
(1–3)	45.66
≥3	42.24
Body mass index (kg/m^2^)	
<25	28.84
(25–30)	39.60
≥30	31.56
Cardiovascular diseases	
No	76.46
Yes	23.54
Hypertension	
No	31.79
Yes	68.21
Diabetes mellitus	
No	79.96
Yes	20.04
Phenotypic age (year)	66.13 ± 0.34
Score of the Digit Symbol Substitution test	47.19 ± 0.80

a Weighted percentage for category variables and weighted mean ± SE for continuous variables.

**Table 2:** Associations between PhenoAge and score of the digit symbol substitution test.

	Crude model^a^		Model 1^b^		Model 2^c^	
	β (95% CI)	*p*-value	β (95% CI)	*p*-value	β (95% CI)	*p*-value
PhenoAge as continuous variable	-0.569 (-0.648, -0.490)	<0.001	-0.599 (-0.684, -0.513)	<0.001	-0.383 (-0.463, -0.303)	<0.001
PhenoAge as category variable						
Q1 (42.24, 58.17)	Reference		Reference		Reference	
Q2 (58.17, 65.36)	-6.043 (-7.925, -4.161)	<0.001	-6.381 (-8.473, -4.289)	<0.001	-4.053 (-5.942, -2.165)	<0.001
Q3 (65.36, 74.26)	-12.813 (-15.286, -10.341)	<0.001	-13.229 (-15.727, -10.730)	<0.001	-8.304 (-10.544, -6.064)	<0.001
Q4 (74.26, 156.58)	-18.489 (-20.767, -16.210)	<0.001	-19.515 (-21.920, -17.110)	<0.001	-12.937 (-15.135, -10.739)	<0.001

CI, confidence interval.

a Crude model: no covariates were adjusted.

b Model 1: sex and race were adjusted.

c Model 2: sex, race, body mass index, education, marital status, poverty status, and chronic disease conditions were adjusted.

### 3.2 Alterations in the microenvironment of PBMC monocytes in different age groups

**Figure 1:**
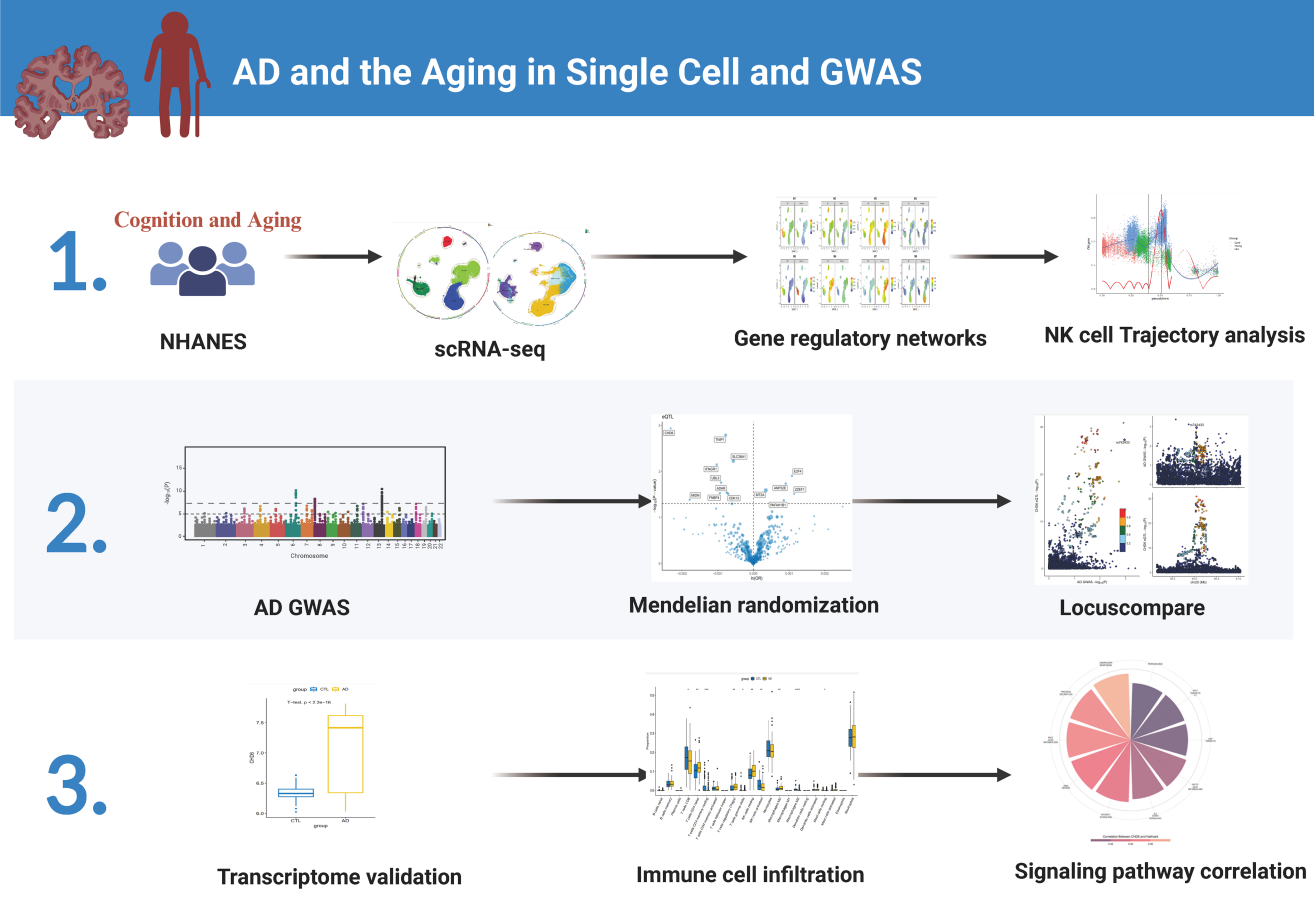
Flowchart. The first step involves the National Health and Nutrition Examination Survey cohort demonstrating the relationship between cognition and aging as well as single-cell analysis of aging and Alzheimer’s disease (AD). The second step entails genome-wide association study analysis of AD. The third step focuses on the transcriptomic validation of key genes’ significance.

**Figure 2:**
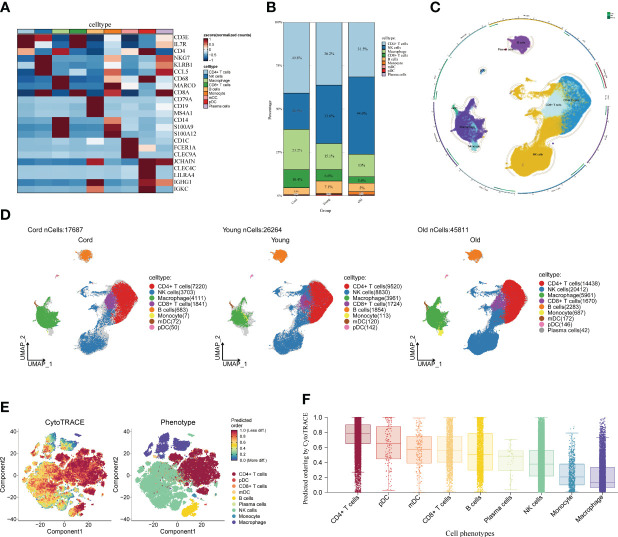
Single-cell analysis of peripheral blood of different age groups. **(A)** Heat map displaying the marker genes of cell subpopulations. The darker the color, the higher the gene expression. **(B)** Bar plot showing the proportions of cell subpopulations in different age groups. The numbers and scale graphs represent the percentage of total cells accounted for. **(C)** Circular plot visualizing cell subpopulations. Different subpopulations of cells are composed of individual points. **(D)** Uniform Manifold Approximation and Projection visualization of cell subpopulation distribution. The distribution of blood cell subpopulations in cord, young, and aging participants are shown separately. **(E)** Evaluation of the differentiation potential of different single-cell subpopulations using CytoTRACE. The darker the color, the higher the predicted dryness score. **(F)** Box plot comparing the differentiation potential of cell subpopulations. The dots and box lines show subgroup scores.

First of all, we drew the research route, which was mainly divided into three steps (**Figure 1**). The analysis was carried out in three main steps. In the first step, the correlation between aging and cognitive dysfunction was demonstrated by a large cohort sample. Moreover, senescence genes in NK cells were identified by blood single-cell sequencing analysis of AD. In the second step, the causal relationship between AD and senescence-related genes in NK cells was demonstrated using MR. In the third step, the expression of key genes was demonstrated by transcriptome sequencing in AD as well as immune cell infiltration in AD and signaling pathways involved in key genes (**Figure 1**). To analyze the cellular immune cell composition of blood at different ages, we next analyzed single-cell sequencing of blood. The heat map in **Figure 2A** shows the expression of marker genes in different cell subsets. The proportion of CD4+ T cells and NK cells increased gradually in cord blood, young participants, and aging participants by comparison of cell ratio histogram. Most of the cells in the aging population are NK cells (44.6%), CD4+ T cells (31.5%), macrophage (13%), CD8+ T cells (3.6%), B cells (1.5%), and monocyte (1.5%) (**Figure 2B**). UMAP visualized the distribution of cell subsets in cord blood (nCells = 17,687), young people (nCells = 26,264), and aging participants (nCells = 45,811) (**Figures 2C, D**).

Furthermore, it demonstrated the importance of NK cells in senescent patients. Stemness differentiation of NK cells was analyzed by using CytoTRACE. CytoTRACE evaluated the differentiation potential of different single-cell subsets and found that CD4+ T cells had the highest differentiation potential (**Figures 2E**, **F**). NK cell subpopulations play a crucial role in aging participants. Therefore, we further investigated the weight relationship between NK cells and other cell subpopulations as well as the signaling pathways that may be influenced. The cell-chat inference of the aging participant showed the number of NK cell interactions or the total interaction intensity (**Supplementary Figures S1A**, **B**). The output signal pathways of NK cells are MIF, ANNEXIN, CCL, IL16, and PARs (**Supplementary Figure S1C**). The hierarchical diagram showed the communication network between NK cells and other subsets of cells in the ANNEXIN, CLL, FTL3, IL16, MIF, and PARs signaling pathways (**Supplementary Figures S1D–I**). By analyzing these communication networks, we can better understand the functional and regulatory roles of NK cells involved in the immune response of aging patients. This was important for an in-depth study of the function and disease development of the aging immune system.

### 3.3 Cell trajectories at different ages

**Figure 3:**
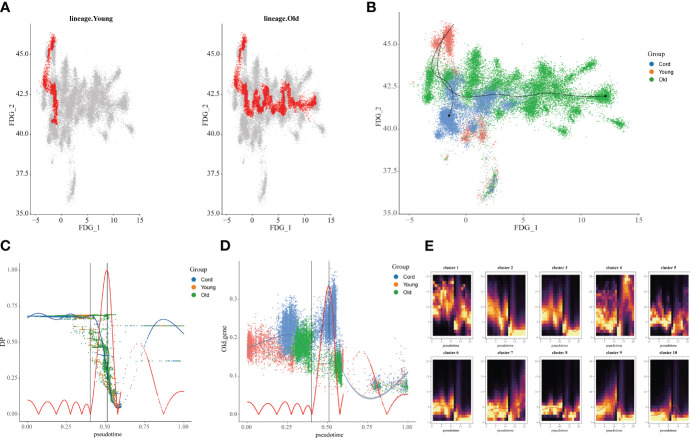
Construction of aging cell trajectories. **(A)** The force-directed graph (FDG) network depicting the trajectories of NK cells in young and elderly populations. The red dots represent the gene expression trajectories visualized. **(B)** Uniform Manifold Approximation and Projection (UMAP) visualization of FDG for cord blood, young individuals, and elderly individuals. The different colored dots represent the cord, young, and aging composition of the trajectory map. **(C)** Relationship between NK cell clusters and differentiation potential. The blue curve represents age-related temporal changes, while the red curve represents changes in differentiation potential. **(D)** UMAP visualization of the temporal changes in the differentiation potential of aging genes. Simulated temporal trajectories of senescence genes at different periods of Cord, young, and aging participants. **(E)** The temporal clustering of aging-related genes by DREVI plots revealed that the differentiation potential of cluster 3 gradually decreased over time. The brighter the color, the denser the expression.

Furthermore, we extracted the NK cell trajectory analysis separately. The diffusion map shows the unique force-directed graph (FDG) of young and aging participants (**Figure 3A**). Moreover, the increase in visualization age was accompanied by the change in trajectory (**Figure 3B**). In addition, the blue curve represents the temporal change in age, and the red curve represents the change in differentiation potential. The aging population NK cell clusters incremented with chronological order, while the differentiation potential began to decline (**Figure 3C**). Looking for key molecular events, aging genes decreased in differentiation potential with increasing temporal order (**Figure 3D**). By DREVI plot temporal clustering, the potential of cluster 3 was found to gradually decrease with time (**Figure 3E**).

### 3.4 Single-cell microenvironmental changes in AD and normal populations

**Figure 4:**
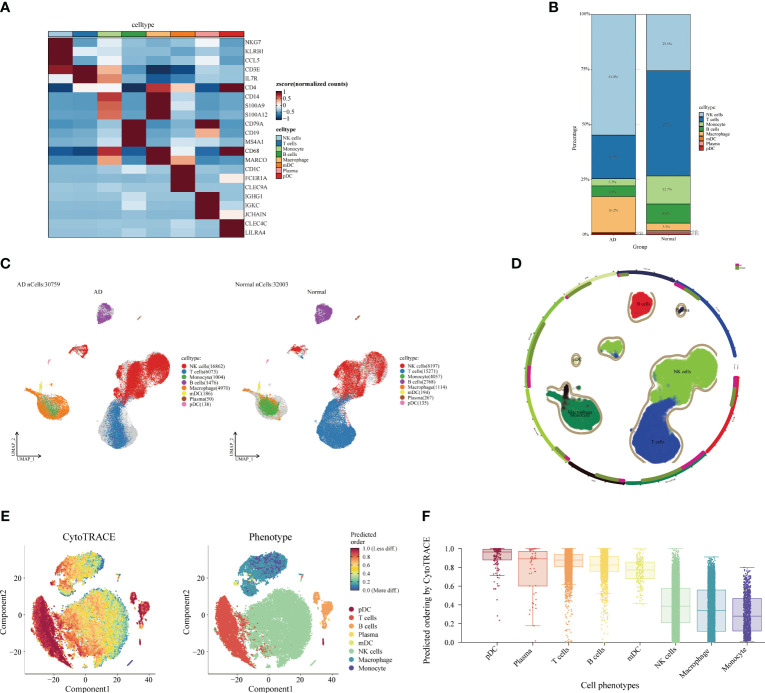
Single-cell analysis of Alzheimer’s disease (AD) and healthy individuals. **(A)** Heat map displaying the marker gene expression of cell subpopulations. The darker the color, the higher the gene expression. **(B)** Bar plot showing the proportions of cell subpopulations in AD and healthy individuals. The percentages represent cell ratios. **(C)** UMAP visualization of cell subpopulation distribution. The colors represent different cell subpopulations. **(D)** Circular plot visualizing cell subpopulations in AD. The colors represent different subpopulations of cells; the more cells, the denser. **(E)** Evaluation of the differentiation potential of AD subpopulations using CytoTRACE. The darker the color, the higher the rating. **(F)** Box plot comparing the differentiation potential of cell subpopulations. The colors represent different subgroups.

Next, we analyzed the cellular and differentiation levels of the cellular microenvironment in blood differing between AD patients and normal patients by single-cell sequencing. A heat map showing the marker genes of PBMC cell subpopulations in AD is presented (**Figure 4A**). The cell histogram scale showed the changes in the proportion of cells in AD *versus* normal patients, where NK cells (54.8%), T cells (19.7%), macrophages (16.2%), B cells (4.8%), and monocytes (3.3%) accounted for the majority of the cells (**Figure 4B**). Moreover, the UMAP visualized the cell subpopulation fractionation in AD (nCells = 30,759) and normal patients (nCells = 32,003) (**Figures 4C**, **D**). To assess the differentiation of immune cells in AD, CytoTRACE assessed the differentiation potential of monocyte subpopulations and found that T cells had a higher differentiation potential (**Figures 4E**, **F**). Moreover, direct interactions of NK cells in AD with other subpopulations were further analyzed. The cell-chat showed that the NK cell has a higher number of interactions or total interaction strength (**Supplementary Figures S2A, B**). The heat map showed that the output signaling pathways of the NK cell subpopulation were MIF and ANNEXIN. The input signaling pathways for the NK cell subpopulation were GALECTI and RESISTIN (**Supplementary Figure S2C**). Hierarchical diagrams showed the communication network of NK cells in the MIF signaling pathway and the ANNEXIN signaling pathway (**Supplementary Figures S2D, E**). The higher number of interactions or total interaction strength observed in NK cells suggests that they are actively involved in the immune response and can modulate the function of other cell populations.

### 3.5 Identifying gene regulation in AD populations

**Figure 5:**
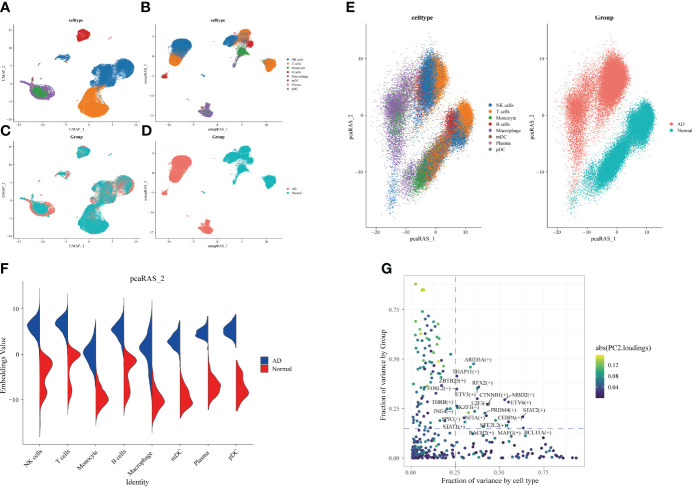
Differences in transcription factor activity between Alzheimer’s disease **(AD)** and healthy individuals. The colors represent different cell subpopulations. **(A)** Uniform Manifold Approximation and Projection (UMAP) visualization of cell subpopulation clustering. **(B)** UMAP visualization of gene expression in AD and healthy individuals. **(C)** Expression of transcription factor activity in different cell subpopulations. **(D)** UMAP visualization of transcription factor activity expression in AD and healthy individuals. **(E)** Visualization of cell subpopulation distribution after PCA dimensionality reduction. Visualization of distribution of AD and healthy individuals after PCA dimensionality reduction. **(F)** Comparison of transcription factor activity in different cell subpopulations between AD and healthy patients. **(G)** Identification of transcription factors with high expression in both grouping and cell types. The horizontal coordinate is the difference in proportions by cell type, and the vertical coordinate is the difference in proportions by group.

**Figure 6:**
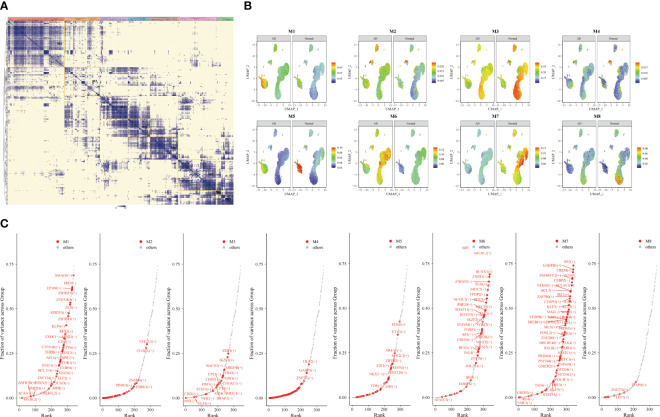
Gene transcription regulation in Alzheimer’s disease (AD). Transcription factor expression clustered into eight modules. **(A)** Identification of different transcription factor clusters through clustering. The darker the color, the higher the transcription factor activity. **(B)** Uniform Manifold Approximation and Projection visualization of transcriptional activity in eight clusters of AD and healthy individuals. **(C)** Top transcription factors in different clusters.

Subsequently, we identified the gene regulatory network of patients through AD blood single-cell sequencing analysis. The UMAP showed the distribution of cellular subpopulations in AD and normal patients (**Figure 5A**). The UMAP showed the distribution of cell subpopulations (**Figure 5B**). The expression distribution of cell subsets in AD patients was almost coincident with that in healthy patients (**Figure 5C**). The UMAP showed the distribution of transcription factors in AD and normal patients (**Figure 5D**). PCA downscaling was performed to analyze transcription factor activity expression, and the AD and normal distributions had a little overlap (**Figure 5E**). Moreover, in most cell subpopulations, AD had a higher transcription factor activity than the normal population (**Figure 5F**). By comparing groups and cell types, ARID3A, THAP11, RFX2, ZBTB25, and MBD2 were found to be some of the high transcription activity factors (**Figure 5G**). Transcription factor clustering was performed and divided into eight subgroups (**Figure 6A**). Compared with the normal population, AD patients had a higher expression of transcription factor activity in the M6 cluster, and most of them were concentrated in NK cells (**Figure 6B**). The expression of the top transcription factors in each cell population was shown separately (**Figure 6C**).

### 3.6 Mendelian randomization and case–control cohort identification of genes that may affect AD

**Figure 7:**
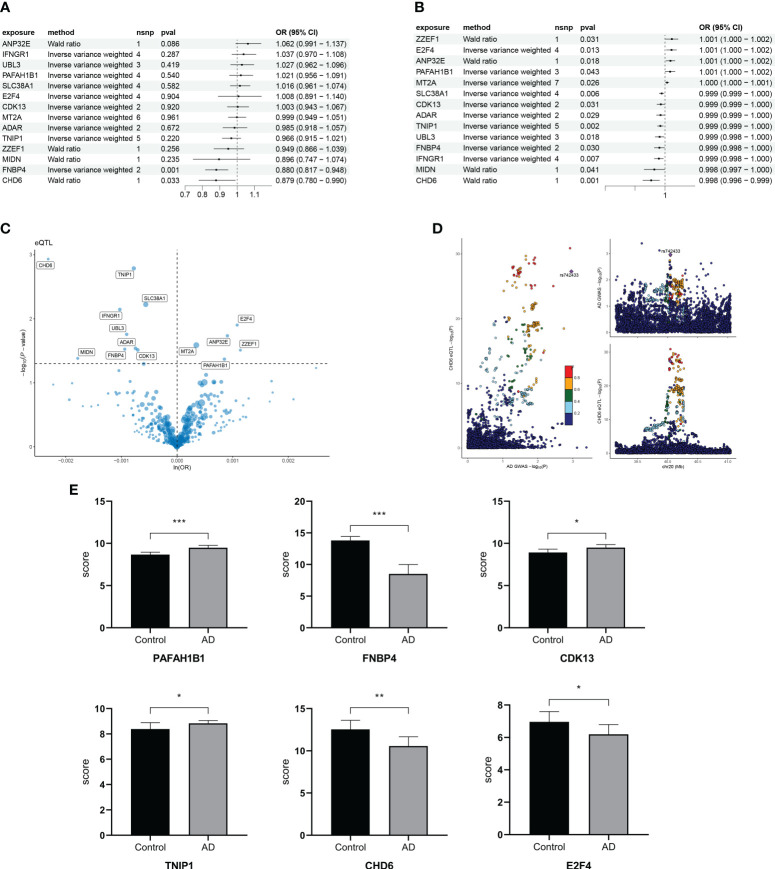
Mendelian randomization analysis of aging trajectory genes and Alzheimer’s disease (AD). **(A)** Forest plot showing the causal correlation between AD and genes. **(B)** Forest plot showing the causal correlation between AD and genes after changing the cohort. **(C)** Volcanic map showing the effect of MR analysis of eQTL gene on the risk of Alzheimer’s disease. **(D)** Display of co-localization analysis of CHD6 gene. The darker the color, the higher the correlation. **(E)** Bar graph comparing the PCR results of key genes in AD and healthy individuals. *p< 0.05, **p< 0.01, ***p< 0.001.

To find the causal relationship between aging-related genes and AD, we next performed an MR analysis. The gene set of cluster 3 after kinetic analysis was used for the subsequent MR analysis (**Supplementary Table S3**). The two-sample MR analysis showed that the expression of *PAFAH1B1*, *IFNGR1*, *CDK13*, *ZZEF1*, *FNBP4*, *SLC38A1*, *UBL3*, *MT2A*, *ANP32E*, *TNIP1*, *ADAR*, *MIDN*, and *E2F4* was potentially associated with the prevalence of AD (**Figure 7A**). After replacing the cohort, *FNBP4* (OR = 0.8801; 95% CI, 0.8173–0.9477; *p* = 0.0007199274) and *CHD6* (OR = 0.8785; 95% CI, 0.7797–0.9899; *p* = 0.0333974294) were found to reduce the risk of AD (**Figure 7B**). The volcano map showed the effect of MR analysis of gene eQTL on the risk of Alzheimer’s disease (**Figure 7C**). The bidirectional Mendelian randomization analysis did not show a causal effect of AD on *FNBP4* and *CHD6* (**Supplementary Table S4**). The Bayesian co-localization showed that *CHD6* (coloc.abf-PPH4 = 0.674) had the same variant as MS (**Figure 7D**). Compared with the normal group, *PAFAH1B1*, *CDK13*, and *TNIP1* were highly expressed in AD patients (*p*< 0.05) (**Figure 7E**). The expression of *FNBP4*, *CHD6*, and *E2F4* was low in AD (*p*< 0.05) (**Figure 7E**).

### 3.7 Verification of *CHD6* expression and immune cell infiltration in the transcriptional group

**Figure 8:**
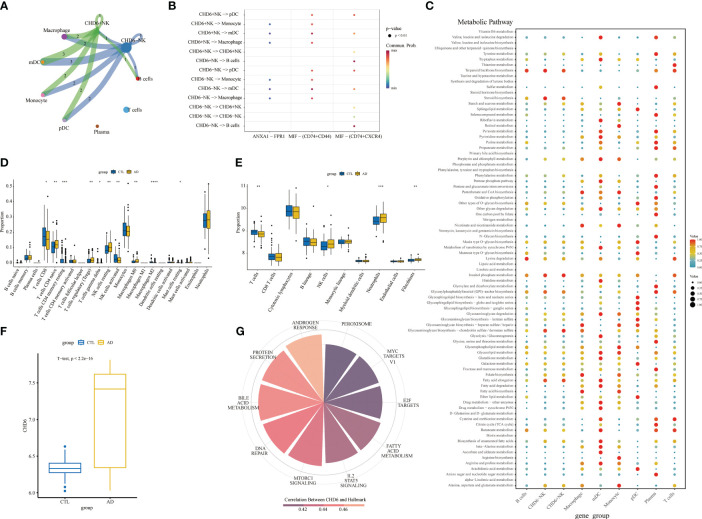
The role of the *CHD6* gene in Alzheimer’s disease (AD). **(A)** Circular plot showing the interaction between *CHD6*+ NK cells and other cell types. **(B)** Bubble plot showing the ligand–receptor relationships between *CHD6*+ NK cells. **(C)** Bubble plot showing the metabolic signaling pathways between *CHD6*+ NK cells and other cell subpopulations. The darker the color, the higher the expression of the signaling pathway. **(D, E)** Box plots comparing immune cell infiltration in AD and healthy individuals. **(F)** Box plot comparing the expression difference of *CHD6* in the transcriptome between normal and AD individuals. **(G)** Correlation of *CHD6* with signaling pathways. The darker the color, the higher the correlation. *p< 0.05, **p< 0.01, ***p< 0.001.

To explore the involvement of *CHD6+* NK cells in the interactions between other immune cells, we performed a cell communication analysis. The cellular communication showed that *CHD6*+ NK cells interacted with most immune cells (**Figure 8A**). *CHD6*+ NK cells communicate cellularly with pDC, monocyte, mDC, and macrophage in the MIF signaling pathway (**Figure 8B**). *CHD6*+ NK cell metabolic signaling pathways were mainly enriched for terpenoid backbone biosynthesis, steroid biosynthesis, inositol phosphate metabolism, glycosaminoglycan biosynthesis chondroitin sulfate/dermatan sulfate, and fatty acid elongation (**Figure 8C**). The CIBERSORT immunocyte infiltration analysis showed that the proportion of CD4 naïve T cells, T cells regulatory, NK cells resting, macrophages M0, and neutrophils in AD was increased (**Figure 8D**). The proportion of CD8+ T cells and NK cells activated in AD decreased (*p*< 0 01) (**Figure 8D**). After a MCPcounter analysis, the proportion of NK cells and neutrophils in AD patients was found to be higher than that in normal people (*p*< 0.05) (**Figure 8E**). This demonstrated the involvement of NK cells as an important immune cell in the disease process of AD patients. The next analysis explored the expression and biological role of CHD6 in AD. We used a transcriptional cohort of AD for the next analysis. In addition, transcriptional sequencing showed that *CHD6* was highly expressed in AD (*p*< 0.001) (**Figure 8F**). The ssGSEA enrichment analysis showed that *CHD6* was associated with androgen response, protein secretion, bile acid metabolism, DNA repair, MTORC1 signaling, IL2 STAT5 signaling, fatty acid metabolism, and E2F target signal pathways (**Figure 8G**).

## 4 Discussion

Alzheimer’s disease is the most common cause of dementia worldwide. With the advent of the aging era, the number of patients with AD is increasing. First of all, we conducted a prospective cohort through the NHANES database to demonstrate the correlation between aging and cognition. At the same time, there have been more and more immunotherapy for AD in recent years. We explored the composition of cellular immune microenvironment in different ages and AD from the single-cell level of PBMC and found that the proportion of NK cells increased with age. By employing transcriptional factor regulation analysis, compared with the normal population, it was found that most of the high transcriptional active factors were concentrated in NK cells of AD. The time sequence of NK cell trajectory in an aging population was constructed by using transcription factors and aging differential genes of NK cells in AD. A cluster with decreased differentiation potential was selected for MR analysis. Finally, we found that *CHD6* may be one of the pathogenic genes affecting AD.

At the same time, our results and previous literature confirm the correlation between cognition and aging (44–46). AD, which is characterized by progressive memory loss and cognitive decline, is thought to account for 60% and 80% of dementia cases (1). In addition, the main risk factor is age (47, 48). Human aging is an inevitable, gradual, whole-biological process. Moreover, some studies have explored the changes in aging-related gene expression in monocytes and other immune cell groups through peripheral blood PBMC transcriptome sequencing (49, 50). Related studies have explored that senescent cells accumulate in aging tissue, which leads to tissue dysfunction (51, 52). Moreover, 1,497 genes were found to be associated with aging (50). At the same time, we also found some NK cell track genes in aging people.

Interestingly, we found that the proportion of NK cells increased in both senescent and AD single-celled microenvironments in our study. NK cells are congenital lymphocytes with dual functions of cytotoxicity and immune regulation and play a key role in the control of malignant tumors and infections (53). NK cells are the core participants in cellular immune monitoring of senescence. With the increase of age, the dysfunction of NK cells is related to the increased burden of infection, malignant tumors, inflammatory diseases, and aging cells (54–56). Aging will seriously affect the immune function of human NK cells. Unlike most immune cells, NK cells increase in number and decrease in function with senescence (57, 58). The possible reason may be the decrease in cytokine secretion and the decrease in cytotoxicity of target cells (59, 60). Senescent cells trigger an immune response, and NK cells eliminate senescent cells through a variety of indirect pathways, such as direct killing and secretion of cytokines or perforin (56). We tried to find the common genes that affect aging and AD. Therefore, we used differential genes in the aging population and genes with higher transcriptional activity in AD. Cell trajectory models were successfully constructed in umbilical cord blood, young people, and aging patients.

The MR is a data analysis method that has gained popularity in recent years in epidemiological etiology research. Its primary objective is to utilize genetic data as a tool to investigate the causal relationship between a specific exposure and an outcome. By performing MR analysis on genes extracted from the aging cell trajectory model, there is a suggestion that *CHD6* could potentially impact AD. The process of cell survival following DNA oxidative damage involves signal transduction, repair mechanisms, and transcriptional events. These processes are often facilitated by nucleosome translocation, exchange, or the action of chromatin remodeling enzymes (61). Unlike other CHD enzymes, *CHD6* is stabilized by reduced degradation during oxidative stress (61). *CHD6* is thought to bind to chromatin in the prostate, expelling nucleosomes from promoters and genomes and transcriptionally activating carcinogenic pathways (62). Moreover, *CHD6* knockdown inhibits cancer cell proliferation, migration, invasion, and tumorigenesis (63). However, there is no in-depth study on the mechanism of nervous system diseases.

This study investigated the relationship between aging and the microenvironment of AD at the single-cell level. However, there are still some limitations that need to be acknowledged. Firstly, it is important to note that our data for this study were obtained exclusively from PBMCs, in contrast to the differential gene analysis performed on tissues. While this approach ensures data consistency, it is essential to consider that the variation in blood cell ratios itself may influence the results. Secondly, this study employed a single-cell data analysis of an Asian population, whereas the GWAS data used was derived from European data. This discrepancy in population origin might potentially diminish the reliability of the findings. Thus, further investigations should be conducted involving populations from other regions. Thirdly, although the NHANES database was utilized to validate the association between cognition and aging using larger populations, additional clinical cohorts should be included for further verification. Further studies with larger sample sizes and more diverse patient populations are necessary for the next steps to validate and extend our findings. Lastly, it is worth acknowledging that there may exist confounding factors or multiple effects in the MR study. Nonetheless, our primary focus remains on establishing causal correlations based on two or more tools, thereby enhancing the reliability of our results. Furthermore, we performed a co-location analysis as a sensitivity analysis to validate the MR results.

## 5 Conclusion

In summary, our study explored AD and aging patients at the cohort, transcriptome sequencing, scRNA-seq, and GWAS levels, providing insights into potential causality. However, further studies are needed to confirm the clinical significance of the relevant experimental results, which may be helpful to guide the diagnosis and treatment of AD in elderly patients.

## Data availability statement

The original contributions presented in the study are included in the article/**Supplementary Material**. Further inquiries can be directed to the corresponding authors.

## Ethics statement

The studies involving humans were approved by the Institutional Ethics Committee and the Institutional Review Board of Liuzhou Workers’ Hospital (Ethics Code: KY2023140). The studies were conducted in accordance with the local legislation and institutional requirements. The human samples used in this study were acquired from primarily isolated as part of your previous study for which ethical approval was obtained. Written informed consent for participation was not required from the participants or the participants’ legal guardians/next of kin in accordance with the national legislation and institutional requirements.

## Author contributions

JL: Conceptualization, Data curation, Formal Analysis, Writing – original draft, Writing – review & editing. YZ: Conceptualization, Data curation, Investigation, Writing – original draft. YY: Conceptualization, Data curation, Validation, Writing – original draft. ZH: Conceptualization, Data curation, Formal Analysis, Writing – review & editing. LW: Investigation, Methodology, Writing – review & editing. CL: Formal Analysis, Project administration, Writing – review & editing. BW: Formal Analysis, Funding acquisition, Project administration, Writing – review & editing. LP: Data curation, Funding acquisition, Methodology, Writing – review & editing. YH: Investigation, Software, Writing – review & editing. YSH: Methodology, Project administration, Writing – review & editing. MY: Data curation, Methodology, Writing – review & editing. ML: Investigation, Methodology, Validation, Writing – review & editing. RL: Writing – review & editing. XY: Data curation, Methodology, Writing – original draft. QL: Methodology, Resources, Writing – review & editing. SD: Methodology, Writing – review & editing, Investigation.

## Supplementary material

Supplementary Figure 1 Cell communication between cell subpopulations in different age groups. **(A, B)** Circular plots displaying the quantity and weight of cell communication. **(C)** Heat map illustrating the input and output signaling pathways of different cell subpopulations. Hierarchical graphs depict the interactions between target cells and ANNEXIN **(D)**, CCL **(E)**, FLT3 **(F)**, IL16 **(G)**, MIF **(H)**, and PARs **(I)**.

Supplementary Figure 2 Cell communication between cell subpopulations in Alzheimer’s disease (AD). **(A, B)** Circular plots displaying the quantity and weight of cell communication. **(C)** Heat map illustrating the input and output signaling pathways of different cell subpopulations in AD. Hierarchical graphs depict the interactions between target cells and MIF **(D)** and ANNEXIN **(E)**.
