# Supplementary figures and images for: Unraveling the mechanisms of NK cell dysfunction in aging and Alzheimer’s disease: insights from GWAS and single-cell transcriptomics
Source: Front Immunol. 2024 Feb 23;15:1360687. doi: 10.3389/fimmu.2024.1360687 (PMC10920339; doi:10.3389/fimmu.2024.1360687)

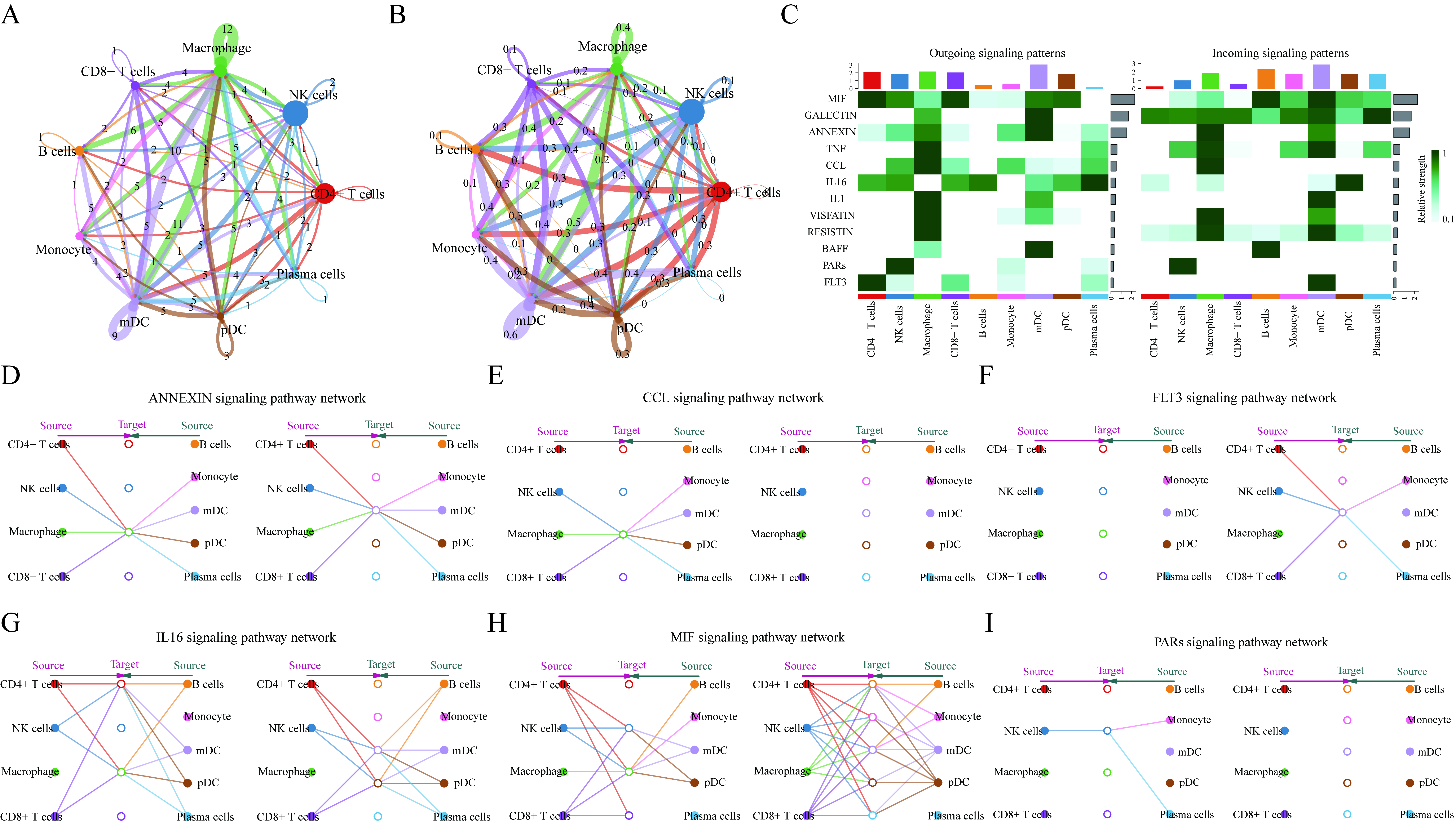

Supplement: Supplementary Figure 1 — Cell communication between cell subpopulations in different age groups. (A, B) Circular plots displaying the quantity and weight of cell communication. (C) Heat map illustrating the input and output signaling pathways of different cell subpopulations. Hierarchical graphs depict the interactions between target cells and ANNEXIN (D), CCL (E), FLT3 (F), IL16 (G), MIF (H), and PARs (I). [file Image_1.tif]

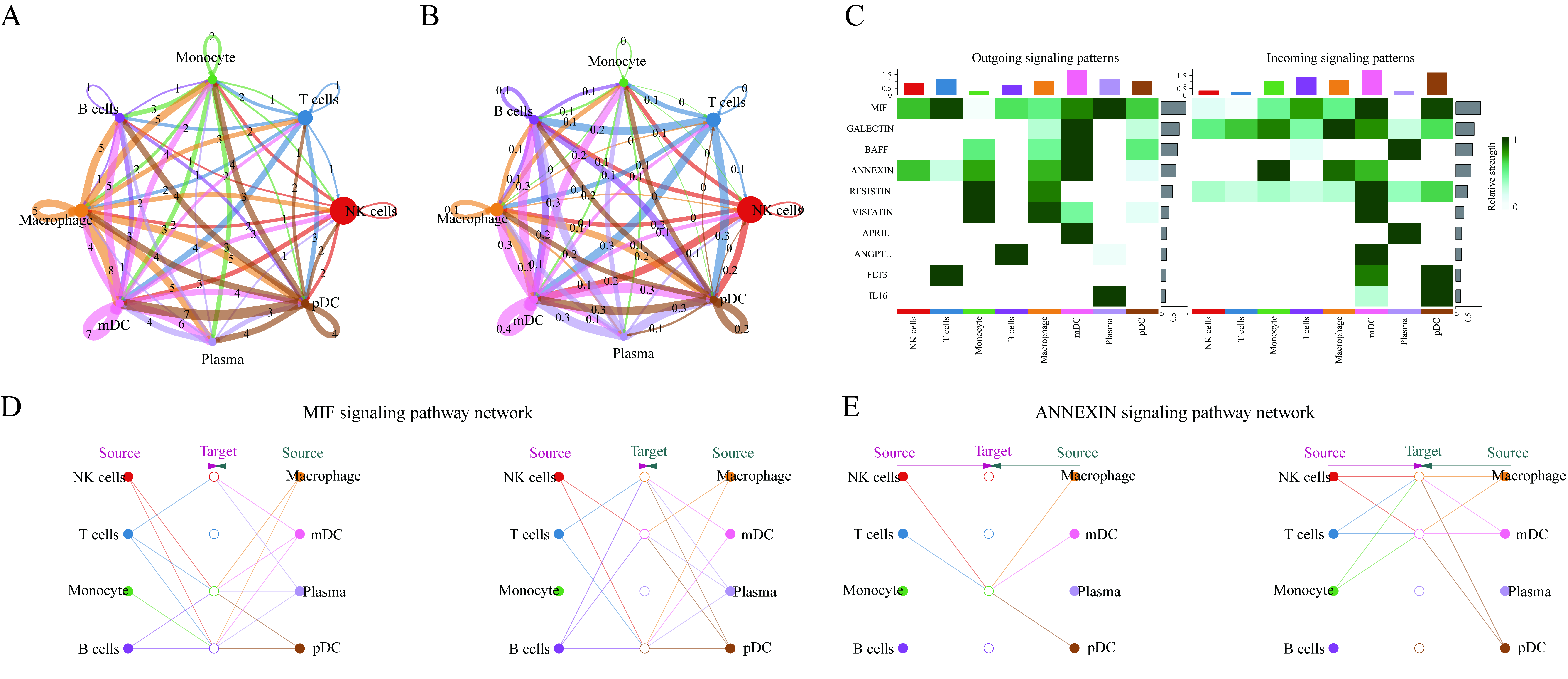

Supplement: Supplementary Figure 2 — Cell communication between cell subpopulations in Alzheimer’s disease (AD). (A, B) Circular plots displaying the quantity and weight of cell communication. (C) Heat map illustrating the input and output signaling pathways of different cell subpopulations in AD. Hierarchical graphs depict the interactions between target cells and MIF (D) and ANNEXIN (E). [file Image_2.tif]
