# Supplementary material for: Unraveling the mechanisms of NK cell dysfunction in aging and Alzheimer’s disease: insights from GWAS and single-cell transcriptomics
Source: Front Immunol. 2024 Feb 23;15:1360687. doi: 10.3389/fimmu.2024.1360687 (PMC10920339; doi:10.3389/fimmu.2024.1360687)
Supplement: Supplementary file 4 [file Table_2.docx]

Table S2. Weighted linear regression of stratified results for associations between PhenoAge and score of the Digit Symbol Substitution Test.

|  | Q1 | Q2 | P-value | Q3 | P-value | Q4 | P-value | P for trend |
| --- | --- | --- | --- | --- | --- | --- | --- | --- |
| Sex |  |  |  |  |  |  |  |  |
| Male | Reference | -2.736( -6.530, 1.057) | 0.150 | -11.939(-15.857, -8.021) | <0.001 | -15.628(-19.063,-12.193) | <0.001 | <0.001 |
| Female | Reference | -8.377(-11.304, -5.451) | <0.001 | -13.11(-16.213,-10.008) | <0.001 | -20.554(-24.452,-16.655) | <0.001 | <0.001 |
| Race/ethnicity |  |  |  |  |  |  |  |  |
| Non-hispanic White | Reference | -6.211( -8.401, -4.021) | <0.001 | -13.176(-16.064,-10.289) | <0.001 | -20.035(-22.563,-17.507) | <0.001 | <0.0001 |
| Non-hispanic Black | Reference | -5.892(-11.654,-0.130) | 0.046 | -9.138(-14.297,-3.979) | 0.002 | -13.731(-20.656,-6.806) | <0.001 | <0.001 |
| Mexican American | Reference | -2.746( -7.519, 2.027) | 0.229 | -8.728(-13.444,-4.012) | 0.002 | -12.234(-17.284,-7.185) | <0.001 | <0.001 |
| Other race/ethnicity | Reference | -10.65(-20.194, -1.106) | 0.033 | -19.225(-28.317,-10.134) | 0.001 | -21.219(-36.539, -5.898) | 0.013 | <0.001 |
| Marital status |  |  |  |  |  |  |  |  |
| Never married | Reference | 0.284(-30.321,30.889) | 0.972 | -0.079(-31.669,31.511) | 0.992 | 3.472(-36.035,42.978) | 0.742 | 0.803 |
| Married/living with partner | Reference | -3.994( -6.268, -1.720) | 0.001 | -13.06(-16.420, -9.700) | <0.001 | -17.883(-20.913,-14.853) | <0.001 | <0.001 |
| Widowed/ divorced | Reference | -9.476(-14.399, -4.552) | <0.001 | -10.83(-15.927, -5.733) | <0.001 | -17.274(-21.531,-13.016) | <0.001 | <0.001 |
| Poverty income ratio |  |  |  |  |  |  |  |  |
| < 1 | Reference | -3.876(-11.730, 3.977) | 0.319 | -8.28(-15.625, -0.935) | 0.029 | -17.982(-25.499,-10.464) | <0.001 | <0.001 |
| [1,3) | Reference | -4.834( -7.817,-1.852) | 0.003 | -9.308(-13.260,-5.355) | <0.001 | -13.245(-16.663,-9.828) | <0.001 | <0.001 |
| ≥ 3 | Reference | -3.445( -5.760, -1.130) | 0.005 | -10.737(-14.790, -6.684) | <0.001 | -15.774(-19.433,-12.115) | <0.001 | <0.001 |
| Education |  |  |  |  |  |  |  |  |
| Below high school | Reference | -2.677( -9.720, 4.366) | 0.441 | -4.246(-10.941, 2.449) | 0.203 | -7.928(-14.753,-1.102) | 0.025 | 0.02 |
| High school | Reference | -5.863( -9.062, -2.663) | <0.001 | -10.373(-14.318, -6.429) | <0.001 | -17.973(-21.135,-14.812) | <0.001 | <0.001 |
| College or above | Reference | -6.136( -8.779, -3.493) | <0.001 | -12.699(-15.208,-10.189) | <0.001 | -17.827(-21.066,-14.587) | <0.001 | <0.001 |
| Body mass index (kg/m^2^) |  |  |  |  |  |  |  |  |
| < 25 | Reference | -7.092(-11.158, -3.027) | 0.001 | -14.995(-19.206,-10.784) | <0.001 | -20.857(-24.933,-16.781) | <0.001 | <0.001 |
| [25, 30) | Reference | -5.9( -8.988, -2.813) | <0.001 | -11.409(-16.200, -6.619) | <0.001 | -16.756(-21.474,-12.039) | <0.001 | <0.001 |
| ≥ 30 | Reference | -5.16( -9.237, -1.082) | 0.015 | -12.43(-16.421, -8.440) | <0.001 | -18.169(-21.530,-14.809) | <0.001 | <0.001 |
| Cardiovascular diseases |  |  |  |  |  |  |  |  |
| No | Reference | -5.569( -8.051, -3.086) | <0.001 | -12.643(-15.023,-10.264) | <0.001 | -17.803(-20.830,-14.777) | <0.001 | <0.001 |
| Yes | Reference | -6.789(-10.550, -3.029) | <0.001 | -11.393(-15.986, -6.800) | <0.001 | -16.444(-20.601,-12.287) | <0.001 | <0.001 |
| Hypertension |  |  |  |  |  |  |  |  |
| No | Reference | -7.348(-10.080, -4.617) | <0.001 | -12.374(-15.183, -9.566) | <0.001 | -17.73(-20.720,-14.741) | <0.001 | <0.001 |
| Yes | Reference | -2.892( -6.271, 0.487) | 0.090 | -12.957(-17.395, -8.519) | <0.001 | -19.389(-23.359,-15.418) | <0.001 | <0.001 |
| Diabetes mellitus |  |  |  |  |  |  |  |  |
| No | Reference | -9.27(-16.889, -1.650) | 0.019 | -12.19(-17.948, -6.432) | <0.001 | -15.383(-19.990,-10.775) | <0.001 | <0.001 |
| Yes | Reference | -4.92( -7.047, -2.793) | <0.001 | -12.436(-15.089, -9.782) | <0.001 | -18.534(-21.271,-15.798) | <0.001 | <0.001 |
